# Identification of NAD^+^ Metabolism-Derived Gene Signatures in Ovarian Cancer Prognosis and Immunotherapy

**DOI:** 10.3389/fgene.2022.905238

**Published:** 2022-06-16

**Authors:** Liang Lin, Li Chen, Zuolian Xie, Jian Chen, Ling Li, An Lin

**Affiliations:** Department of Gynecology, Fujian Medical University Cancer Hospital, Fujian Cancer Hospital, Fuzhou, China

**Keywords:** NAD^+^, ovarian cancer, prognostic signature, biomarker, immunotherapy

## Abstract

**Background:** Nicotinamide adenine dinucleotide (NAD^+^) has emerged as a critical regulator of cell signaling and survival pathways, affecting tumor initiation and progression. In this study it was investigated whether circulating NAD^+^ metabolism-related genes (NMRGs) could be used to predict immunotherapy response in ovarian cancer (OC) patients.

**Method:** In this study, NMRGs were comprehensively examined in OC patients, three distinct NMRGs subtypes were identified through unsupervised clustering, and an NAD^+^-related prognostic model was generated based on LASSO Cox regression analysis and generated a risk score (RS). ROC curves and an independent validation cohort were used to assess the model’s accuracy. A GSEA enrichment analysis was performed to investigate possible functional pathways. Furthermore, the role of RS in the tumor microenvironment, immunotherapy, and chemotherapy was also investigated.

**Result:** We found three different subgroups based on NMRGs expression patterns. Twelve genes were selected by LASSO regression to create a prognostic risk signature. High-RS was founded to be linked to a worse prognosis. In Ovarian Cancer Patients, RS is an independent prognostic marker. Immune infiltrating cells were considerably overexpressed in the low-RS group, as immune-related functional pathways were significantly enriched. Furthermore, immunotherapy prediction reveal that patients with low-RS are more sensitive to immunotherapy.

**Conclusion:** For a patient with OC, NMRGs are promising biomarkers. Our prognostic signature has potential predictive value for OC prognosis and immunotherapy response. The results of this study may help improve our understanding of NMRG in OCs.

## Background

Ovarian cancer (OC) is the deadliest gynecological cancer with few initial symptoms and a poor prognosis (Webb and Jordan, 2017; Kossaï et al., 2018; Matulonis, 2018). It is the fifth leading cause of cancer-related death in women, and fewer than 50% of women survive beyond 5 years after diagnosis due to the rapid emergence of chemoresistance coupled with the lack of effective early detection strategies. A number of cancers, including OC, have recently been treated with immunotherapy, although OC patients are highly heterogeneous and some are immune to immunotherapy (Roett and Evans, 2009; Ottevanger, 2017). Furthermore, OC has a high probability of recurrence and medication resistance (Tew, 2016; Sipos et al., 2021). Therefore, a great deal of research is required to advance understanding of disease etiology, identify risk factors, and develop early detection methods and effective molecular biomarkers.

It is believed that metabolic reprogramming plays a role in the genesis of tumors. NAD^+^ plays a key role in maintaining cellular homeostasis, genome stability, cell growth, cell death, and immune responses (Newman and Maddocks, 2017; Pramono et al., 2020; Navas and Carnero, 2021). In cells, NAD exists in two states: oxidized (NAD^+^) and reduced (NADH). NAD^+^ stimulates cancer cell growth by enhancing anaerobic glycolysis *via* glyceraldehyde 3-phosphate dehydrogenase (GAPDH) and lactate dehydrogenase (LDH). Most of exhibit increased ratios of NAD^+^/NADH and NADP^+^/NADPH, implying that NAD^+^ plays a significant role in cancer (Nacarelli et al., 2020; Ghanem et al., 2021; Wang et al., 2022). In addition, NAD^+^ acts as a substrate of sirtuins, PARPs, and cADPRSs in many different signaling pathways, including DNA repair, inflammatory responses, posttranslational modifications, senescence, and apoptosis (Sultani et al., 2017; Rajman et al., 2018; Zapata-Pérez et al., 2021). Due to the ineffectiveness of traditional anticancer therapies, researchers are seeking new therapeutic targets. In this context, NMRGs could be a potential new target. By investigating the role of NMRGs in OC, new treatments can be developed and a better understanding of the disease can be gained.

Bioinformatics techniques have made it possible for researchers to study OC in greater detail in recent years. The primary objective of this study is to create NMRG signals that could provide insights into clinical treatment and prognosis for patients with OC. Based on the expression levels of NMRGs, we divided OC patients into two subgroups using The Cancer Genome Atlas (TCGA) and Gene Expression Omnibus (GEO) databases. Furthermore, we constructed a prognostic model of OC patients based on NMRGs and generated an RS. We also examined the model’s stability and the significance of RS in clinical therapy. In summary, we successfully developed a risk model for NAD^+^ that could be used in clinical therapy and diagnostics.

## Materials and Methods

### Ovarian Cancer Data Source and Preprocessing

We retrieved RNA expression and clinical data from ovarian cancer patients (Supplementary Table S1) in The Cancer Genome Atlas (TCGA) and The Genotype-Tissue Expression (GTEx) databases. Normal tissue/paracancerous tissue of OC in GTEx was used as control. Tissues from patients with GSE26193 (Supplementary Table S2) were used as the validation dataset. The GSE26193 annotation file is available at Affymetrix Human Genome U133 Plus 2.0 Array (HG-U133_Plus_2). We converted Fragments per kilobase (FPKM) values to transcripts per million (TPM) for the TCGA cohort. Patients with missing survival information were excluded from the study. The SVA package of the R software is used to correct for the effects of batch processing on data. We used the KEGG database (Pathway: hsa00760) and the Reactome database (R-HSA-196807) (Supplementary Table S3) to obtain NMRGs (Li C. et al., 2022). The Immune Checkpoint Immunophenoscore (IPS) is a good predictor of patient response to CTLA-4 and PD-1 immunotherapy. The Cancer Immunome Atlas (TCIA) provided immunophenotyping score files for immune checkpoint inhibitor (ICI) patients.

### Ovarian Cancer Analysis Based on Online Database

Metascape (http://metascape.org/gp/) is a gene-annotation and analysis tool commonly used in genetic research. (Zhou et al., 2019; Han et al., 2021; Ye et al., 2021). The Metascape database was used to perform Gene Ontology (GO) and Kyoto Encyclopedia of Genes and Genomes (KEGG) pathway enrichment analyses NMRGs.

The Cancer Genome Project (CGP, https://cancer.sanger.ac.uk/cosmic) is one of the most comprehensive databases exploring the impact of somatic mutations in human cancer. We analyzed the tumor mutational status of Ovarian Cancer based on COSMIC (Jubb et al., 2018; Sondka et al., 2018).

### Construction and Verification of NAD^+^ Metabolism-Related Genes Signatures

We performed unsupervised consensus clustering to elucidate the relationship between NAD^+^ metabolic subtypes and prognosis. We used the R package “ConsensuClusterPlus” and repeated 1,000 times to guarantee the stability of the clustering (Wang et al., 2020; Wu et al., 2021a). Using the consensus clustering approach, determine the optimal numbers of clusters. Significant DEGs are present in several subtypes, and they were subjected to univariate Cox regression analysis to further screen DEGs linked with OC prognosis. After that, these genes were subjected to LASSO regression analysis to find more useful prognostic factors. Finally, 12 genes strongly connected to OS, and an RS was generated for each OC patient based on the expression levels of these genes and the Cox regression coefficient (Cao et al., 2020; Liang et al., 2020; Zhao et al., 2021). According to the median risk score, OC patients were divided into high-risk and low-risk subgroups. The prognostic prediction performance can evaluate using Kaplan-Meier survival analysis and time-dependent ROC curves. The validation cohort for the model was GSE26193. Cox regression analysis, both univariate and multivariate, was used to see if RS may be an independent prognostic factor in OC patients.

### GSVA Enrichment Analysis

We used the “GSVA” R software tool to perform GSVA enrichment analysis to learn more about the differences in functional pathways and biological processes between distinct subtypes and high- and low-RS groups. For functional annotation, the R package “cluster profile” was used, and the gene set file (c2. cp.kegg.v7.2. symbols.gmt) was obtained from the MSigDB database (https://www.gsea-msigdb.org) (Sun et al., 2020; Chen L. et al., 2021; Wu et al., 2021b).

### Tumor Microenvironment Analysis

The “ESTIMATE” package was used to predict the composition of the immune stroma in the tumor microenvironment (TME) of Ovarian Cancer patients, as well as to calculate Immune Score, Stromal Score and ESTIMATE Score (Fan et al., 2021; Li Y. et al., 2022). The ssGSEA algorithm was used to quantify dissimilarities in immune cell infiltration subsets and immune function enrichment between high- and low-RS groups. ssGSEA is a popular enrichment algorithm extensively utilized in medical studies (Liu et al., 2021; Liu et al., 2022a; Liu et al., 2022b; Liu et al., 2022c).

### Statistical Analysis

All statistical analyses are performed by the use of R version 4.1.2. ifferentially expressed genes (DEGs) were identified using the R package “limma,” and survival analysis was performed using the “survival” and “survminer” packages (Ritchie et al., 2015). The “ggplots” software was used to create the volcano and heatmaps. The IC_50_ of chemotherapeutic medicines was predicted using the “pRRophetic” software (Geeleher et al., 2014; Wang et al., 2021). All statistical studies used two-sided, and *p* < 0.05 was considered to be significant.

## Results

### Identification and Functional Enrichment Analysis of NAD^+^ Metabolism-Related Genes

**FIGURE 1:**
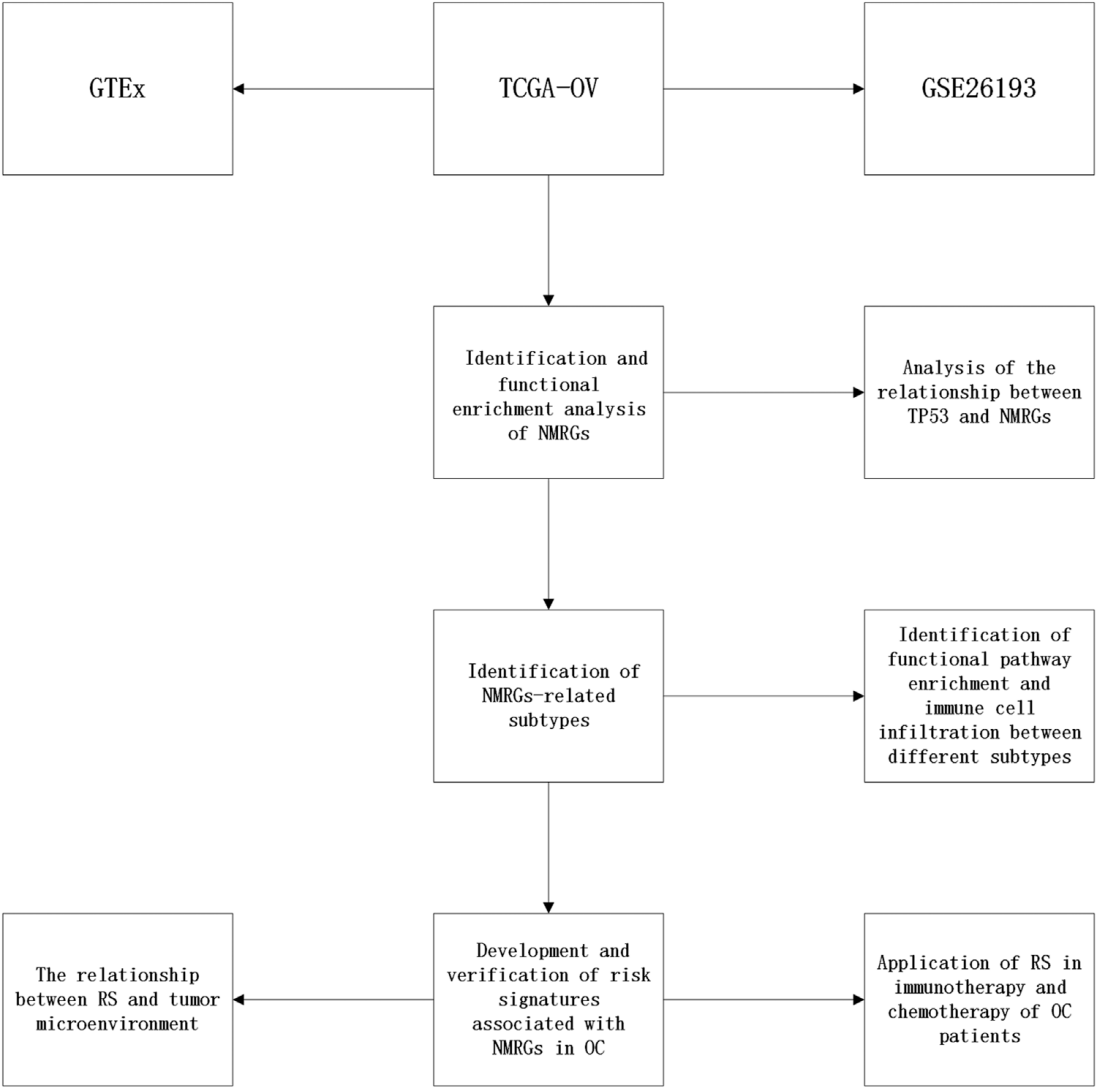
Flowchart of this study.

**FIGURE 2:**
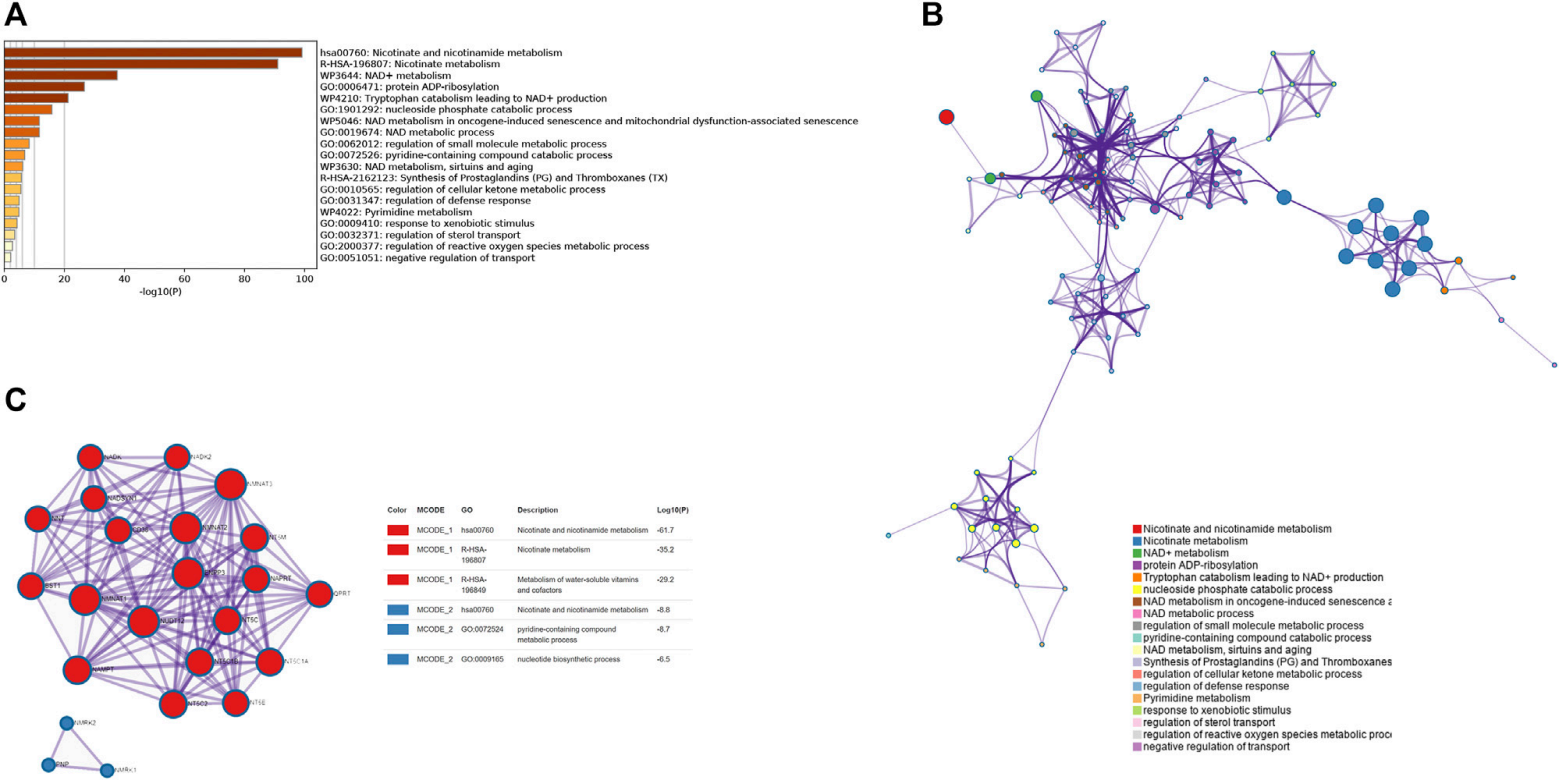
Functional enrichment of NMRGs and visualization of interactome analysis results. **(A)** Metascape enrichment analysis for the NMRGs. **(B)** Metascape enrichment network visualization showing the intra-cluster and inter-cluster similarities of enriched terms. **(C)** Metascape visualization of the interactome network formed by NMRGs candidates, where the MCODE complexes are colored according to their identities.

Figure 1 depicts the study’s analysis process. The prognostic research revealed that most NMRGs were strongly linked with OC prognosis, implying that NMRGs play a key role in OC (Supplementary Figure S1). We performed a functional enrichment analysis of NMRGs using the Metascape database and found that they were significantly enriched in metabolism-related available pathways, including columns Nicotinate and nicotinamide metabolism, Nicotinate metabolism, NAD^+^ metabolism, NAD metabolic process, regulation of small molecule metabolic process, regulation of cellular ketone metabolic process, Pyrimidine metabolism, and regulation of reactive oxygen species metabolic activities (Figure 2A). Figure 2B illustrates the link between enrichment pathways. In addition, we identified the regulatory networks of crucial proteins in NMRGs using protein interaction enrichment analysis, and we discovered that they were mainly connected with nicotinate and nicotinamide metabolism, nicotinate metabolism, metabolism of water-soluble vitamins and cofactors, nicotinate and nicotinamide metabolism, pyridine-containing compound metabolic process, and nucleotide biosynthetic process (Figure 2C). Another important finding was that most NMRGs were dysregulated in OC. *NAXE*, *RNLS*, *PNP*, *NT5DC4*, *PARP9*, *NMNAT2*, *RDH14*, *CD38* were significantly higher expressed in OC compared to normal tissues, while *NAXD*, *AOX1*, *PAPR6*, *SLC5A8*, *NT5C*, *ENPP1*, *NADSYN1*, *SIRT2*, *PTGIS*, *NT5C2*, *NMRK1*, *NMNAT3* were significantly lower expressed in OC (Supplementary Figure S2).

### Analysis of the Relationship Between TP53 and NAD^+^ Metabolism-Related Genes

**FIGURE 3:**
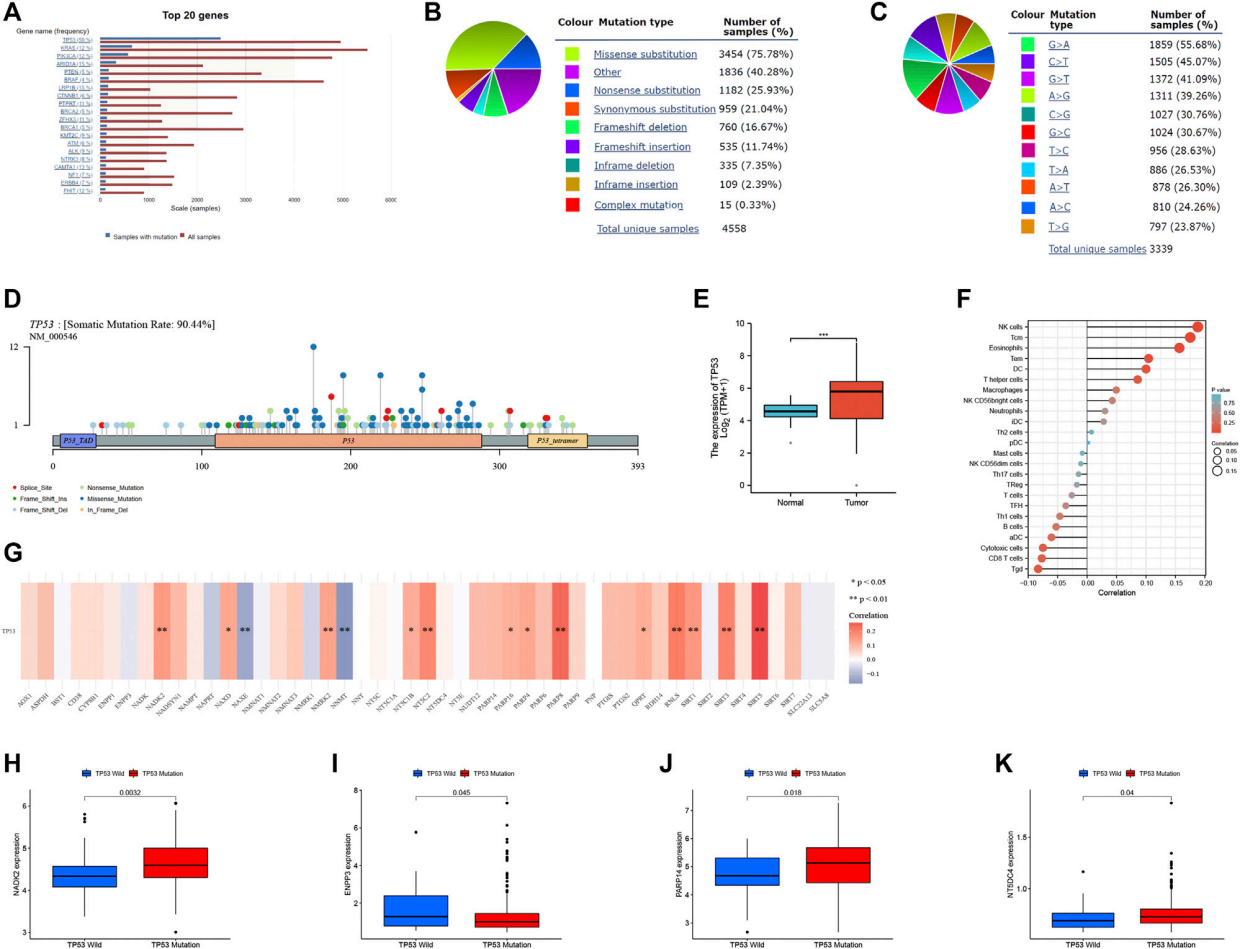
Analysis of the relationship between *TP53* and NMRGs. COSMIC database analysis of OC mutation distributions **(A)** and its types **(B, C)**. **(D)** Lollipop charts of the mutated *TP53* gene, the figure caption shows the somatic mutation rate, and the subheadings shows the name of somatic mutation. **(E)** *TP53* was significantly overexpressed in the tumor group. **(F)** The relationship between *TP53* and immune infiltrating cells. **(G)** Relationship between *TP53* and NMRGs. **(H–K)** Differences in the expression levels of NMRGs between the *TP53* mutant group and the wild-type group. *p < 0.05, **p < 0.01, ***p < 0.001.

Based on the COSMIC database, we looked at the mutation status of OC (Figure 3A) and discovered that missense substitution and G > A mutations were most common (Figures 3B,C). We also provide a lollipop plot of the distribution of mutations in the *TP53* gene based on the TCGA data, as *TP53* is the gene with the highest mutation frequency in OC (Figure 3D). *TP53* was also strongly expressed in OC tissues (Figure 3E) and had a significant positive link with several immune-infiltrating cells such as NK cells, TCM, and Eosinophils (Figure 3F). Furthermore, we analyzed the relationship between *TP53* and NMRGs, we found that *TP53* was positively correlated with *NADK*, *NAXD*, *NMRK2*, *NT5C2*, *NT5C1B*, *PARP16*, *PARP4*, *PARP8*, *QPRT*, *RNLS*, *SIRT1*, *SIRT3*, *SIRT5*, and with *NAXE*, *NNMT* has a negative correlation (Figure 3G). Further study found that the *TP53* mutant group tended to have higher *NADK2* (Figure 3H), *PARP14* (Figure 3J), *NT5DC4* (Figure 3K) expression, and lower *ENPP3* (Figure 3I) expression.

### Identification of NAD^+^ Metabolism-Related Genes-Related Subtypes in Ovarian Cancer Patients

**FIGURE 4:**
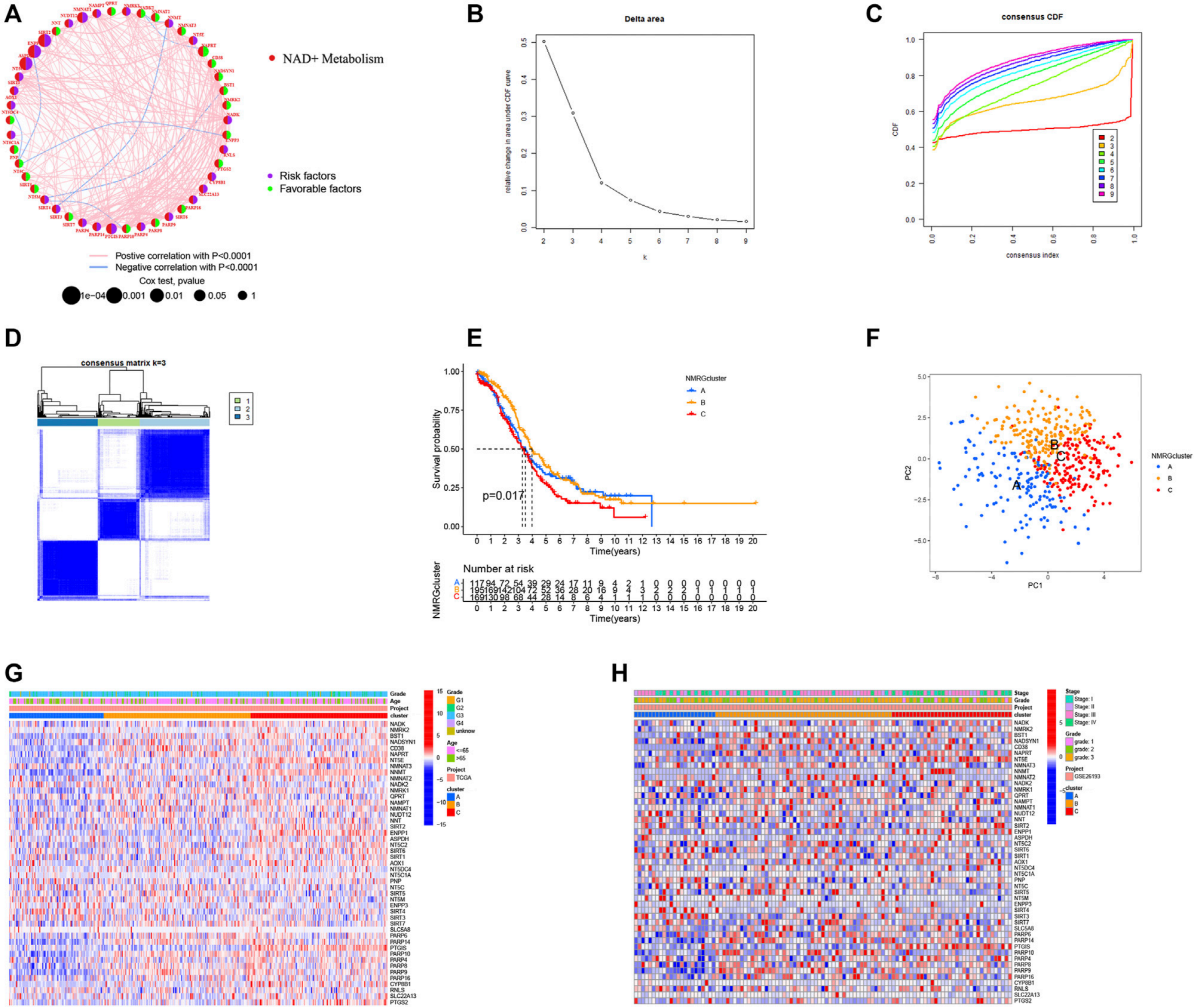
Identification of NMRGs-related subtypes in OC patients. **(A)** A risk network for NMRGs. **(B)** Consensus clustering cumulative distribution function (CDF) for *k* = 2 to 9. **(C)** Relative change in area under the CDF curve for *k* = 2 to 9. **(D)** *K* = 3 was a relatively stable distinction of the samples in the OC dataset. **(E)** Kaplan-Meier survival curve showing the relationship between NMRGs-related subtypes and overall survival. **(F)** Principal component analysis (PCA) analysis of NMRGcluster. The heatmap shows the clinical characteristics of different subtypes of TCGA **(G)** and GEO **(H)** patients.

NMRGs have long been thought to have a crucial function in OC. For further analysis, we created NMRG’s risk network by combining the OC patient data from the TCGA and GEO databases into one cohort with the batch correction to remove differences between the data. Findings revealed that most NMRGs show positive correlation relationships and may be risk factors for OC (Figure 4A). The “Consensus Cluster Plus” R software was used to classify OC patients based on NMRG expression level (Figures 4B–D). The best stable clustering result came from this analysis when *k* = 3. We discovered three distinct subgroups: NMRG cluster A, NMRG cluster B, and NMRG cluster C, respectively. According to the predictive analysis results, patients with NMRG cluster C had a considerably worse outcome (*p* = 0.017; Figure 4E). PCA analysis revealed that the NMRG clusters were divided into three discrete clusters (Figure 4F). The heatmap also depicts the clinical characteristics of several subgroups of TCGA (Figure 4G) and GEO (Figure 4H) patients (Supplementary Table S4). Furthermore, we found that patients with NMRG cluster C had higher *TP53* mutation frequencies and lower *TP53* expression levels (Supplementary Figure S3).

### Identification of Functional Pathway Enrichment and Immune Cell Infiltration Between Different Subtypes

**FIGURE 5:**
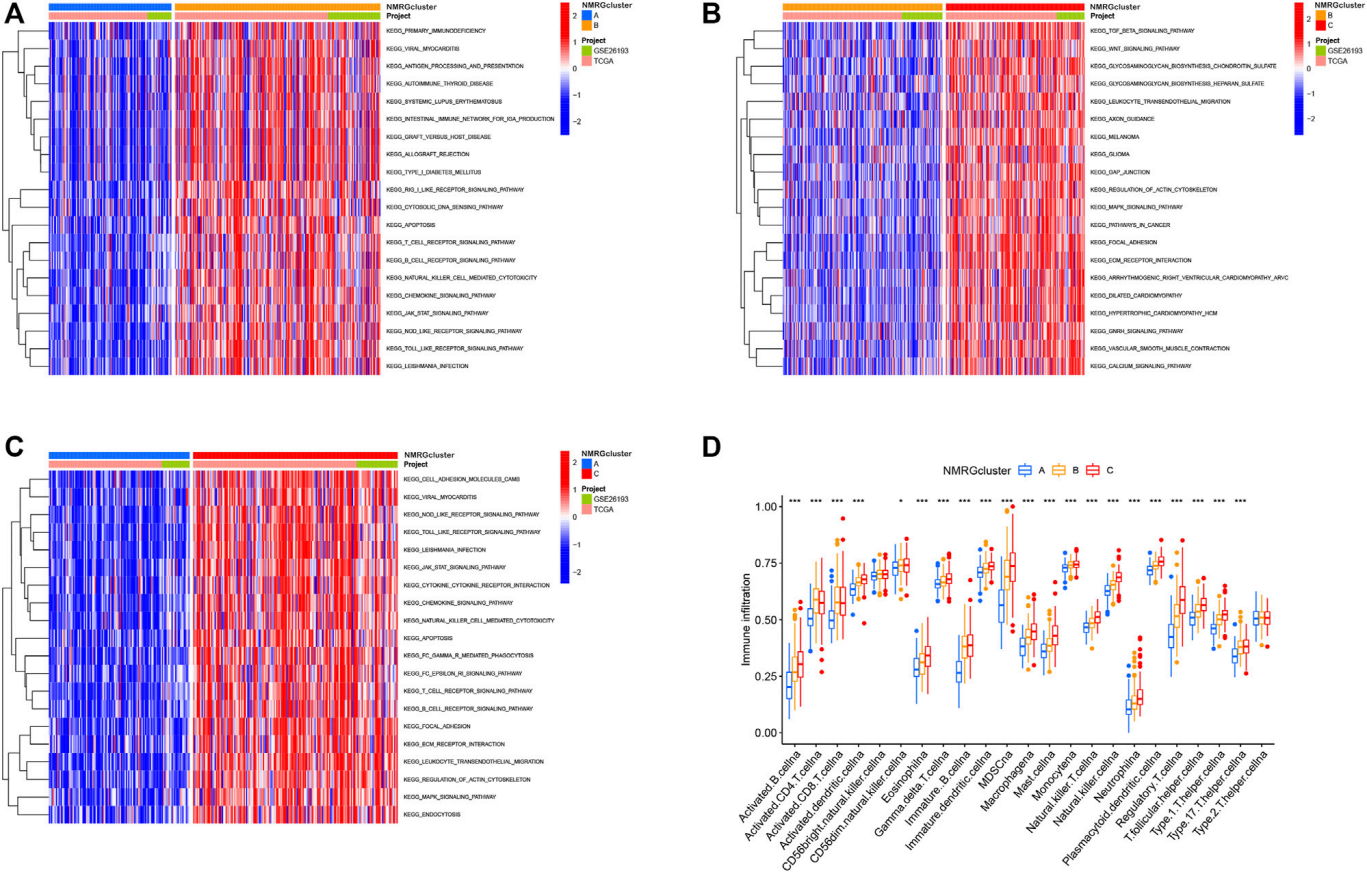
Identification of functional pathway enrichment and immune cell infiltration between different subtypes. **(A–C)** GSVA enrichment analysis shows the activation states of biological pathways in different subtypes. The heat map was used to visualize these biological processes, and red represented activated pathways and blue represented inhibited pathways. **(D)** Tumor microenvironment analysis of NMRGcluster subtypes. *p < 0.05, **p < 0.01, ***p < 0.001.

Results of GSVA enrichment analysis (Figure 5A) depicted that NMRGcluster B was mainly enriched in apoptosis and signaling related pathways, such as RIG I like receptor signaling pathway, Cytosolic DNA sensing pathway, Apoptosis, Antigen processing and presentation, T cell receptor signaling pathway, B cell receptor signaling pathway, JAK STAT signaling pathway, NOD like receptor signaling pathway, and Toll-like receptor signaling pathway. According to Figures 5B,C, TGF beta signaling route, Wnt signaling pathway, Melanoma, Glioma, Cancer pathways, Focal adhesion, JAK STAT signaling pathway, T cell receptor signaling pathway, B cell receptor signaling pathway, Mark signaling pathway were prominent in NMRG Cluster C. Furthermore, the intricacy of immune cell infiltration among the three species subtypes was revealed by ssGSEA enrichment analysis. Immune cell infiltration was lowest in NMRG cluster A. Most of the immune cells, such as activated B cells, activated dendritic cell, CD56dim natural killer cell, Eosinophilia, Gamma delta T cell, Immature B cell, and Immature dendritic cell, were abundant in NMRG cluster C (Figure 5D).

### Development and Verification of Risk Signatures Associated With NAD^+^ Metabolism-Related Genes in Ovarian Cancer

**FIGURE 6:**
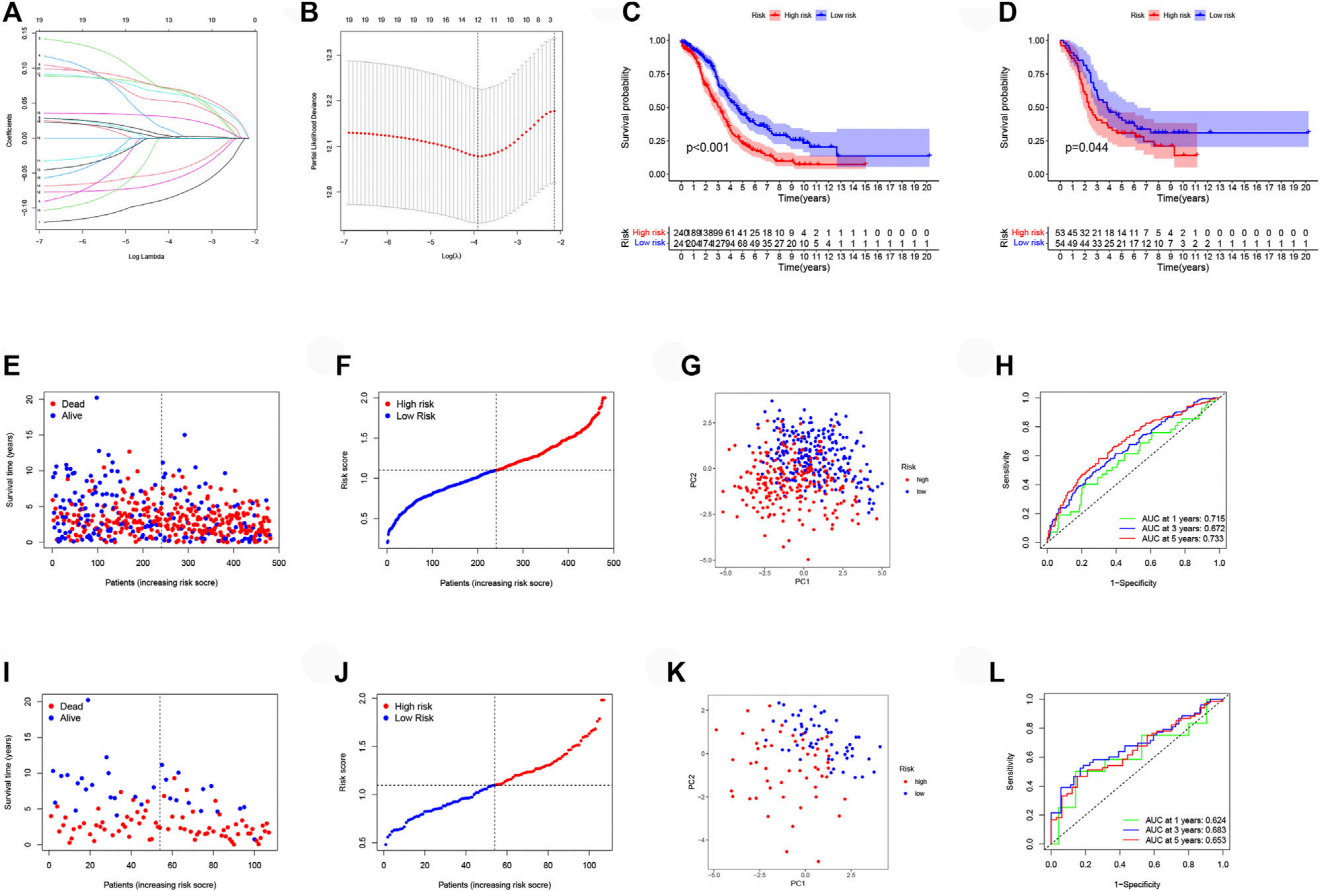
Development and verification of risk signatures associated with NMRGs in OC. **(A)** Cross‐validation for tuning parameter selection in the lasso regression. **(B)** Validation was performed for tuning parameter selection through the least absolute shrinkage and selection operator (LASSO) regression model for overall survival (OS). **(C)** Training cohort, Kaplan-Meier survival analysis of high and low RS subgroups. **(D)** Validation cohort, Kaplan-Meier survival analysis of high and low RS subgroups. **(E)** Training cohort, patient’s survival status. **(F)** Training cohort-RS distribution of patients. **(G)** Training cohort-PCA analysis. **(H)** Training cohort-plots of the AUC for time-dependent ROC performance. **(I)** Validation cohort, patient’s survival status. **(J)** Validation cohort-RS distribution of patients. **(K)** Validation cohort-PCA analysis. **(L)** Validation cohort-plots of the AUC for time-dependent ROC performance.

We discovered 91 shared genes across the 3 categories to further investigate the association between NMRGs-related subtypes and prognosis (Supplementary Figure S4; Supplementary Table S5). Univariate COX analysis was performed on TCGA data to screen genes associated with prognosis. The LASSO regression method was used to further develop the OC prognostic model and establish a risk score (RS). Finally, risk signatures for 12 genes were discovered (Figures 6A,B). The risk score is calculated as follows: RS = (−0.083 * *CXCL11* exp.) + (0.070 * *VSIG4* exp.) + (0.009 * *MS4A7* exp.) + (0.002 * *SULF1* exp.) + (0.052 * *SIRPA* exp.) + (0.069 * *RARRES1* exp.) + (−0.059 * *IGHG1* exp.) + (−0.047 * *PIGR* exp.) + (0.063 * *ZFP36* exp.) + (0.029 * *OGN* exp.) + (0.001 * *MXRA8* exp.) + (−0.070 * *FBLN2* exp.).

According to the median value of RS, OC patients were divided into low-risk group and high-risk group, and the cut-off value was 1.102, that is, patients with RS greater than 1.102 were in the high-risk group, and those with RS less than 1.102 were in the low-risk group. The GSE26193 cohort was used as the validation cohort and its RS was evaluated in the same way. The training cohort (*p* < 0.001; Figure 6C) and the validation cohort (*p* = 0.044; Figure 6D) showed that patients with high RS had a significantly worse prognosis. The patient’s survival status (Figures 6E,I) and risk distribution were also explored (Figures 6F,J). The PCA analysis revealed that RS has a more remarkable ability to separate patients into two classes (Figures 6G,K). The AUCs of the training cohort at years 1, 3, and 5 were 0.715, 0.672, and 0.733, respectively (Figure 6H), and the AUCs of the validation cohort at years 1, 3, and 5 were 0.624, 0.683, and 0.653, respectively, confirming the model’s stability (Figure 6L). The prognostic Nomogram plot analysis results revealed that RS was a good predictor of OC patient’s prognosis (Supplementary Figure S5). The results of univariate and multivariate COX analysis of TCGA and GEO data further indicated that RS was an independent prognostic factor in patients with OC (Supplementary Figure S6).

### The Relationship Between Risk Score and Tumor Microenvironment

**FIGURE 7:**
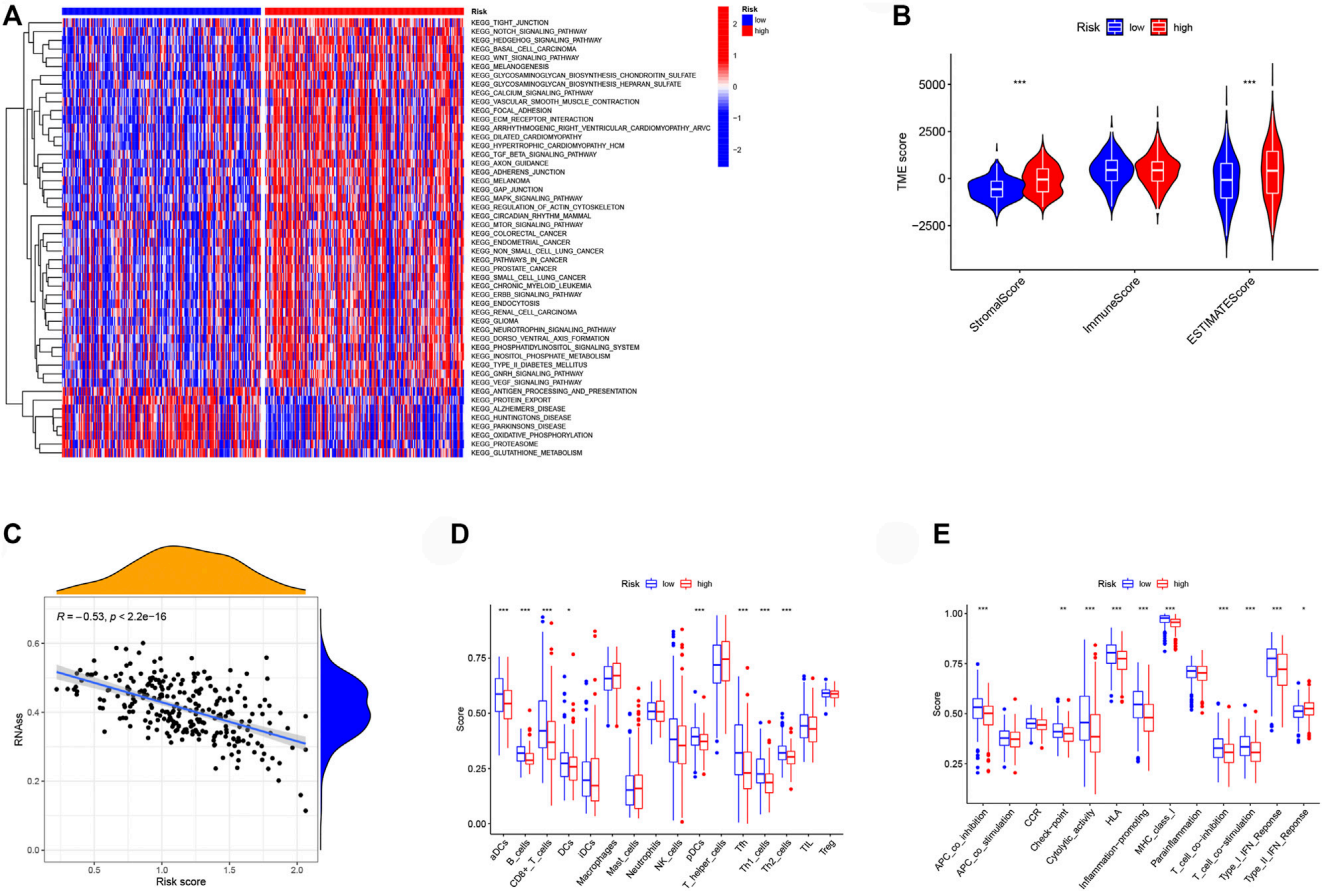
The relationship between RS and tumor microenvironment. **(A)** GSVA enrichment analysis shows the activation states of biological pathways in different subtypes. The heat map was used to visualize these biological processes, and red represented activated pathways and blue represented inhibited pathways. **(B)** Comparison of TME scores between low- and high-risk groups. **(C)** The relationship between RS and RNAss. **(D)** Comparison of the infiltration of 16 immune cells between low- and high-risk group. **(E)** Comparison of the immune functions between low- and high-risk group. *p < 0.05, **p < 0.01, ***p < 0.001.

We discovered enhanced functional pathways between high- and low-RS groups to investigate further the applicability usefulness of our created RS in Ovarian Cancer. The high-RS group was found to be significantly associated with several cancer-related pathways, including colorectal cancer, endometrial cancer, non-small cell lung cancer, pathways in cancer, prostate cancer, small cell lung cancer, chronic myeloid leukemia, erbb signaling pathway, renal cell carcinoma, glioma, wnt signaling pathway, notch signaling pathway (Figure 7A). This result further revealed that patients in the high-risk group had a poor prognosis, multiple cancer-regulated pathways were enriched in the high-risk group, and different cancers may have crosstalk between NMRGs. The high-RS group had a higher stromal score and estimate score (Figure 7B), and was adversely connected with tumor stemness, according to study (Figure 7C). Furthermore, the results of immune cell infiltration analysis revealed that the high RS group had lower immune infiltrating cell enrichment and immune function pathways, such as aDCs, B cells, CD8^+^ T cells, DCs, NK cells, APC co inhibition, Checkpoint, Cytolytic activity, HLA, and Inflammation promoting gene (Figures 7D,E).

### Application of Risk Score in Immunotherapy and Chemotherapy of Ovarian Cancer Patients

**FIGURE 8:**
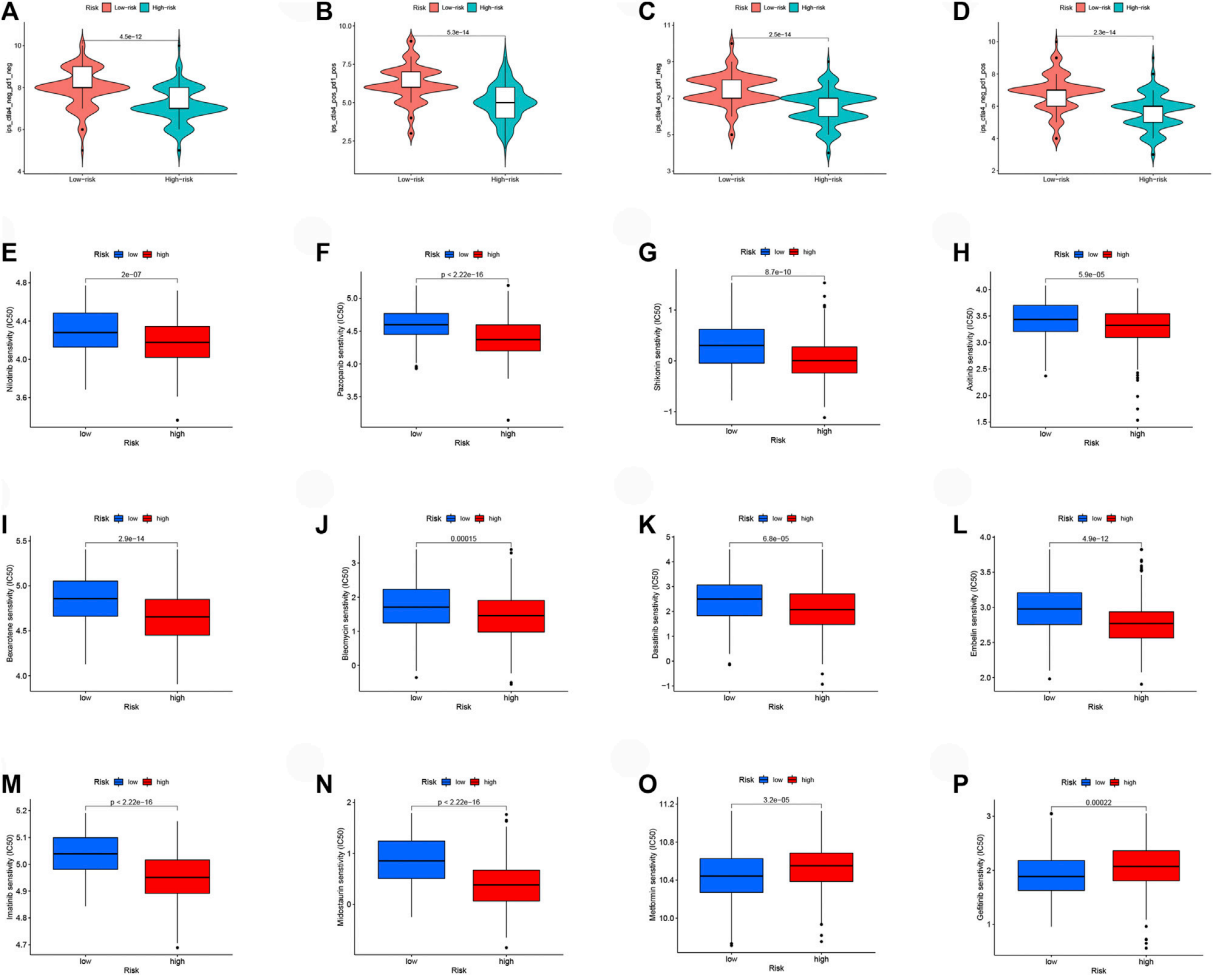
Application of RS in immunotherapy and chemotherapy of OC patients. Immunotherapy in patients with high- and low-RS groups **(A)** CTLA4− PD1−; **(B)** CTLA4+ PD1+; **(C)** CTLA4+ PD1−; **(D)** CTLA4− PD1+. Analysis of drug sensitivity in high- and low-RS groups **(E–P)**.

The potential NMRGs-related RS to predict the prognosis of OC patients has been demonstrated. We gathered immunotherapy data of OC patients from the TCIA database to further enhance the clinical application value of RS, and we discovered that patients with low-RS tend to have higher IPS scores, are more responsive to immune checkpoint blockade therapy (PD1/CTLA4), and may have superior efficacy (Figures 8A–D). The TIDE algorithm further validated our conclusion that patients in the low-risk group were more sensitive to immunotherapy (Supplementary Figure S7). In addition, we compared the IC_50_ of common chemotherapeutic drugs in high and low-RS patients, and found that, except for Metformin (Figure 8O), most drugs had lower IC_50_ scores in high-RS patients, indicating high-RS patients were more susceptible to these drugs (Figure 8E–N), except for Metformin (Figure 8O), Gefitinib (Figure 8P).

## Discussion

OC is one of the most dangerous gynecological cancers, with a significant mortality rate. Despite improvements in OS survival rates over the past 30 years, the 10-year survival rate for most patients remains low (Wu et al., 2020; Yang et al., 2020; Morand et al., 2021). Early symptoms of OC are subtle, and there are no reliable prognostic markers. As a coenzyme of redox reaction in the cytoplasm and mitochondria, NAD^+^ is essential for most basic biological functions in the cell (Li et al., 2019; Sharif et al., 2019; Palavalli Parsons et al., 2021). Although there is growing evidence that individuals with OC have altered NAD^+^ metabolism-related molecules or chemicals, no research on the NAD^+^ metabolic signature of OC prognosis have been reported (Fang et al., 2015; Chen J. et al., 2021; Challa et al., 2021; Valabrega et al., 2021).

In this study, we used public databases to gather OC expression profile data and comprehensively examined the involvement of NMRGs in OC. The majority of the NMRGs were show to be significantly linked with the prognosis of OC. *TP53* is a well-known tumor suppressor that plays a critical function in cell cycle regulation (Schuijer and Berns, 2003; Vitale et al., 2020). We discovered that *TP53* has a high mutation frequency in OC that *TP53* expression levels were connected with the expression levels of multiple NMRGs, highlighting the necessity of investigating NMRGs even more. We divided OC patients into three subtypes based on NMRG expression levels, with the NMRGcluster C subtype having the highest chance of survival. In addition, using the LASSO regression analysis method, we built a predictive model combining 12 genes based on the differential genes between the three subtypes, which was confirmed in the GEO dataset. RS was an independent predictor of OC patients in both univariate and multivariate Cox regression analyses. The tumor microenvironment study revealed that RS may be used to characterize the tumor microenvironment of OC patients, with patients with high-RS having poor prognosis and decreased immune-infiltrating cells enrichment. We also discovered that RS might be used to guide clinical treatment and patients with low-RS are more likely to respond to immunotherapy. The results of the medication sensitivity study between high- and low-RS groups were also helpful in treating OC patients.

In this study, we developed a model that contains 12 NMRG signatures, which could help in the prognosis and clinical treatment of OC patients. We acknowledge, however, that our research has some limitations. The ROC results of the validation cohort were low, and the model may have certain errors in predicting the prognosis of some OC patients. In addition, further *in vitro* and *in vivo* experiments are required to validate our results, especially the model’s prediction of response to immunotherapy and chemotherapy.

## Conclusion

Overall, we identified a new prognostic NMRGs signature of OC patients. This signature may help to develop new OC molecular targets and explore more effective immunotherapy strategies.

## Data Availability

The original contributions presented in the study are included in the article/Supplementary Material, further inquiries can be directed to the corresponding authors.

## Supplementary Material

Supplementary Figure S1 Prognostic analysis of NMRGs in OC.

Supplementary Figure S2 The expression distribution of NMRGs in tumor tissues and normal tissues. ****p* < 0.001.

Supplementary Figure S3
*TP53* level and mutation relationship with NMRGcusters. **P* < 0.05, ***P* < 0.01, ****P* < 0.001.

Supplementary Figure S4 91 shared genes found in 3 categories.

Supplementary Figure S5 Prognostic nomogram analysis.

Supplementary Figure S6 Univariate **(A)** and multivariate **(B)** COX analysis of TCGA data. Univariate **(C)** and multivariate **(D)** COX analysis of GEO data.

Supplementary Figure S7 Potential ICB response was predicted with TIDE algorithm. ****p* < 0.001.

Supplementary Table S1 Clinical information in patients with TCGA-OV. unknow: data missing/unavailable.

Supplementary Table S2 Clinical information in patients with GSE26193.

Supplementary Table S3 NAD^+^ metabolism-related genes.

Supplementary Table S4 Clustering characteristics of patients.

Supplementary Table S5 91 shared genes found in 3 categories.

## Abbreviations

OC, ovarian cancer; TCGA, the cancer genome atlas; GEO, gene expression omnibus; NAD+, nicotinamide adenine dinucleotide; NMRG, NAD+ metabolism-related gene; RS, risk score; ICI, immune checkpoint inhibitor; TME, tumor microenvironment; LASSO, least absolute shrinkage and selection operator; GTEx, genotype-tissue expression; GO, gene ontology; KEGG, kyoto encyclopedia of genes and genomes; DEGs, differentially expressed genes; PCA, principal component analysis.
