# Supplementary figures and images for: Identification of NAD+ Metabolism-Derived Gene Signatures in Ovarian Cancer Prognosis and Immunotherapy
Source: Front Genet. 2022 Jun 16;13:905238. doi: 10.3389/fgene.2022.905238 (PMC9243463; doi:10.3389/fgene.2022.905238)

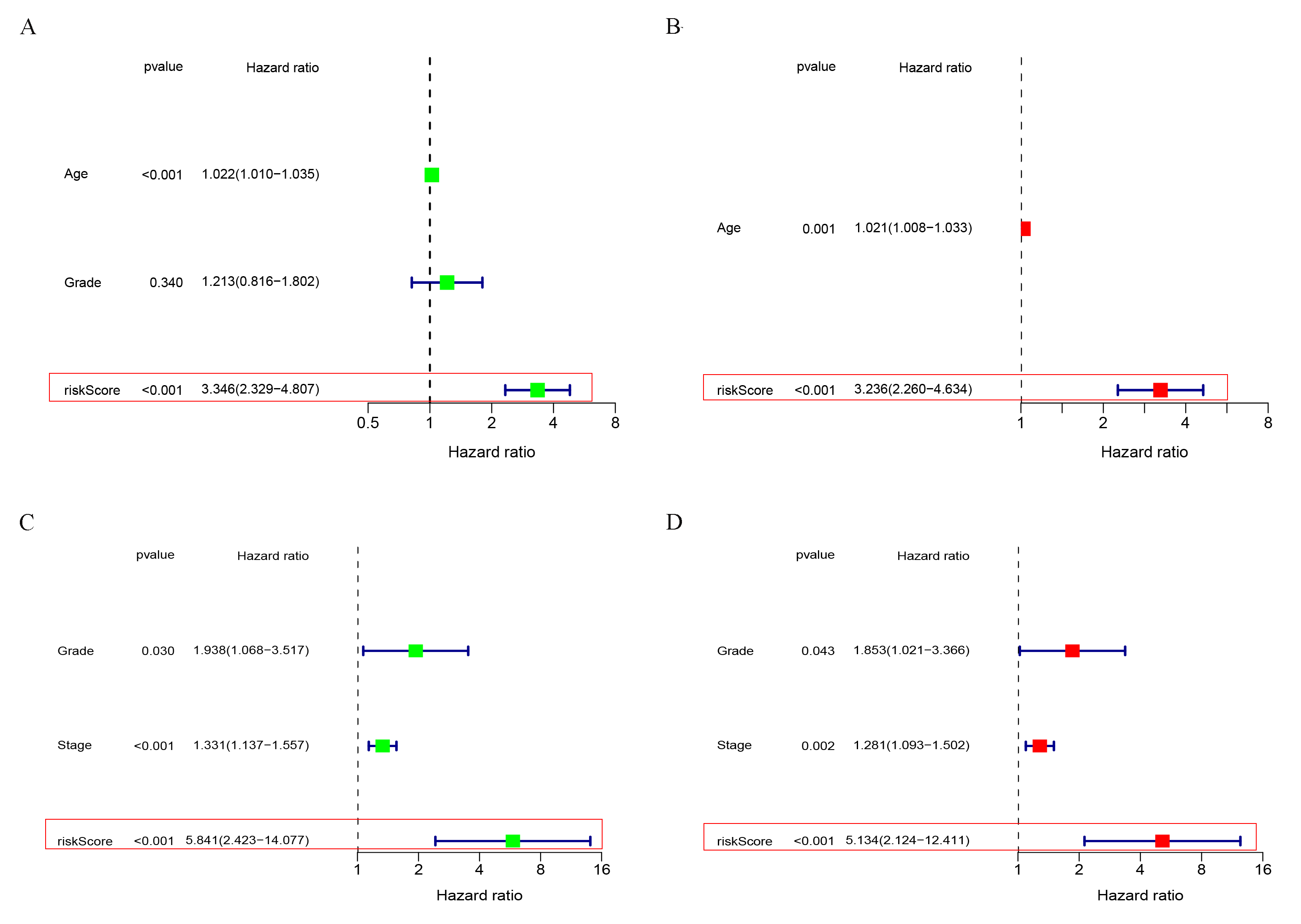

Supplement: Supplementary file 1 [file Image6.TIF]

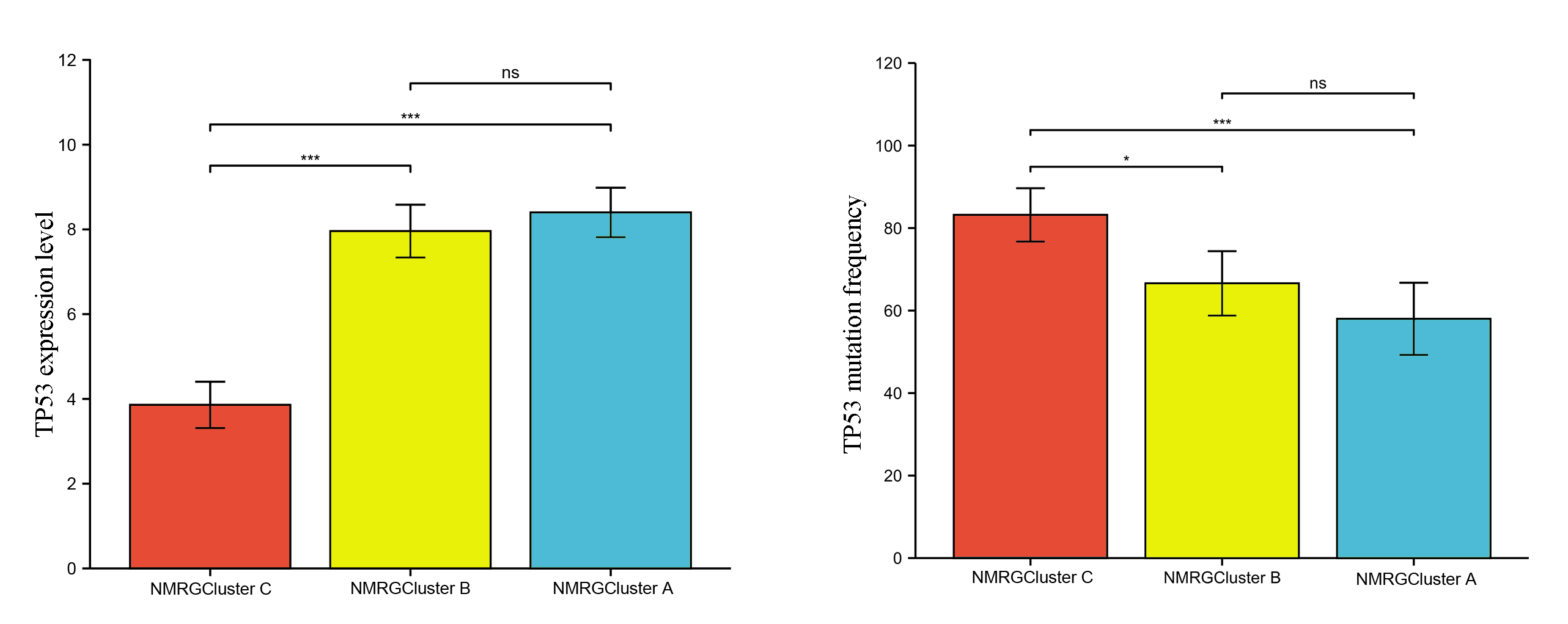

Supplement: Supplementary file 2 [file Image3.TIF]

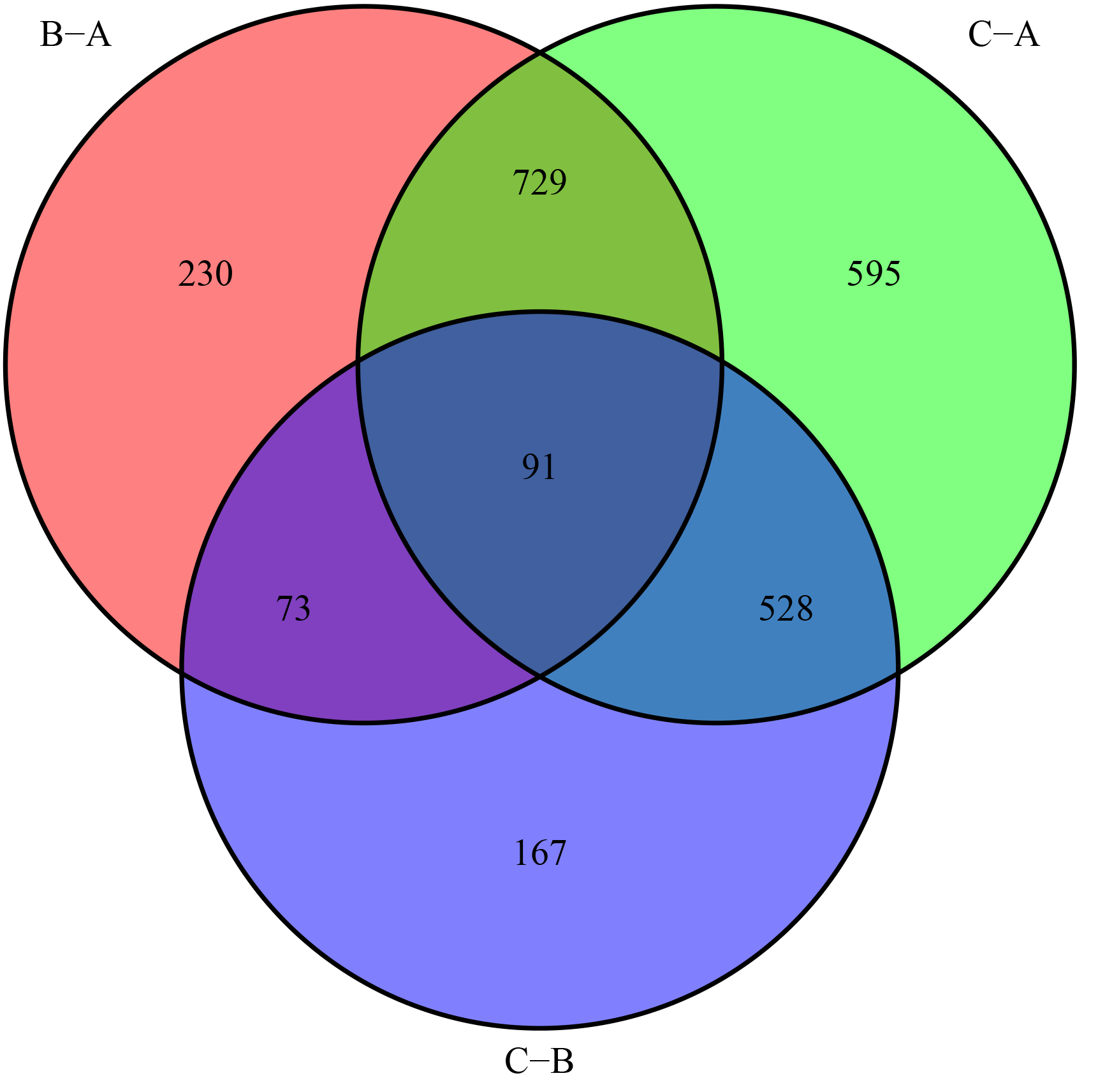

Supplement: Supplementary file 3 [file Image4.TIF]

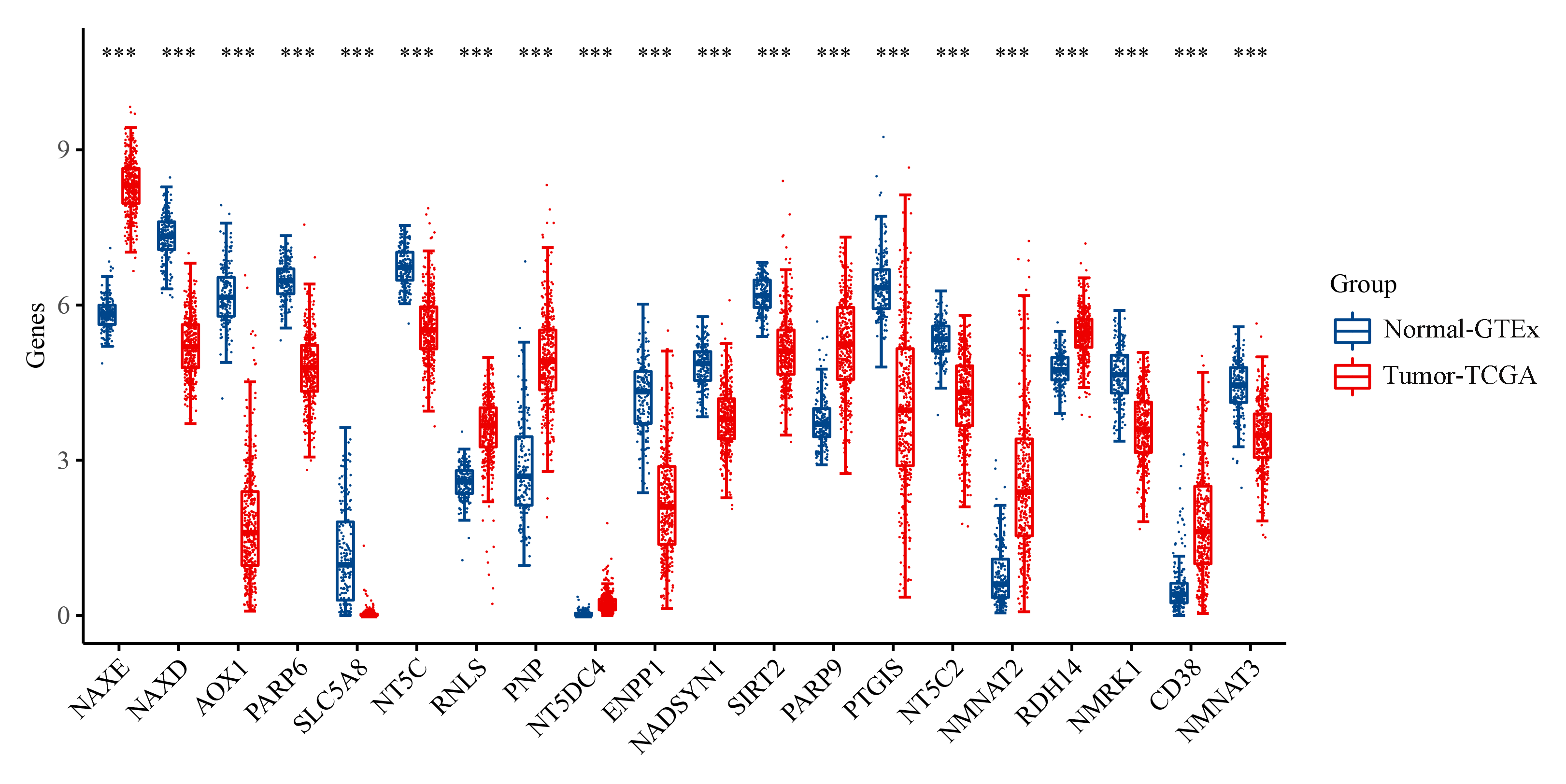

Supplement: Supplementary file 5 [file Image2.TIF]

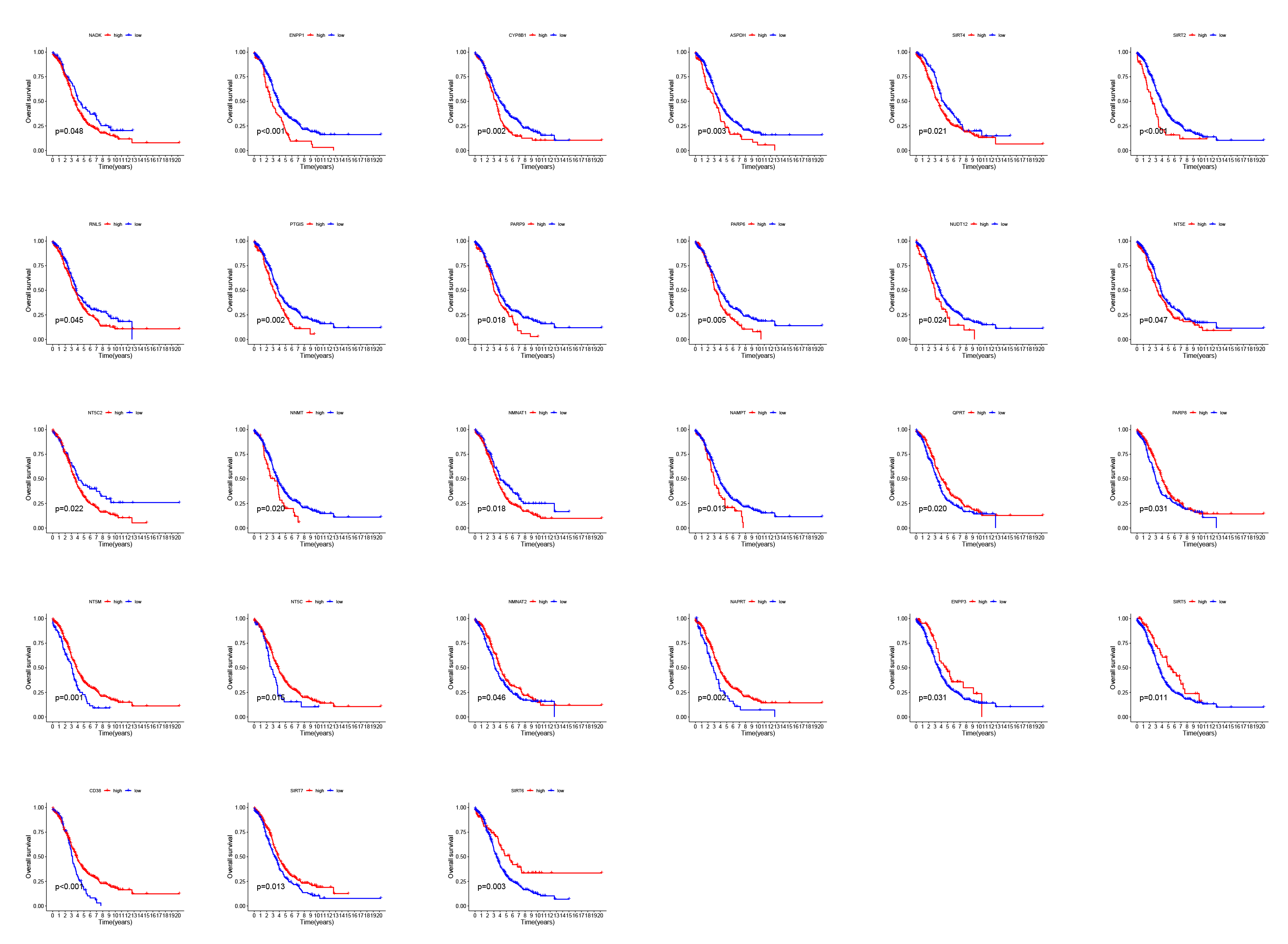

Supplement: Supplementary file 6 [file Image1.TIF]

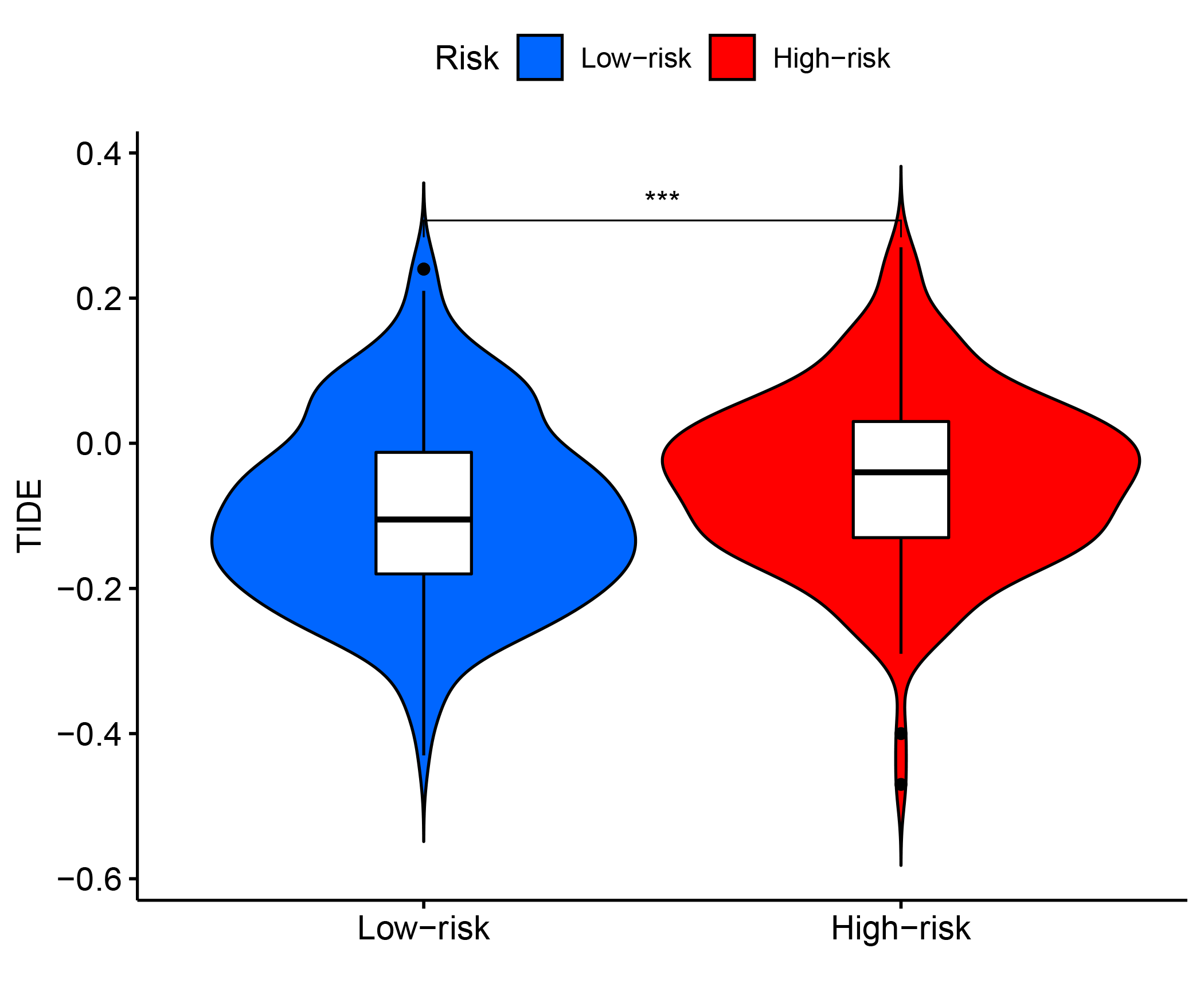

Supplement: Supplementary file 7 [file Image7.TIF]

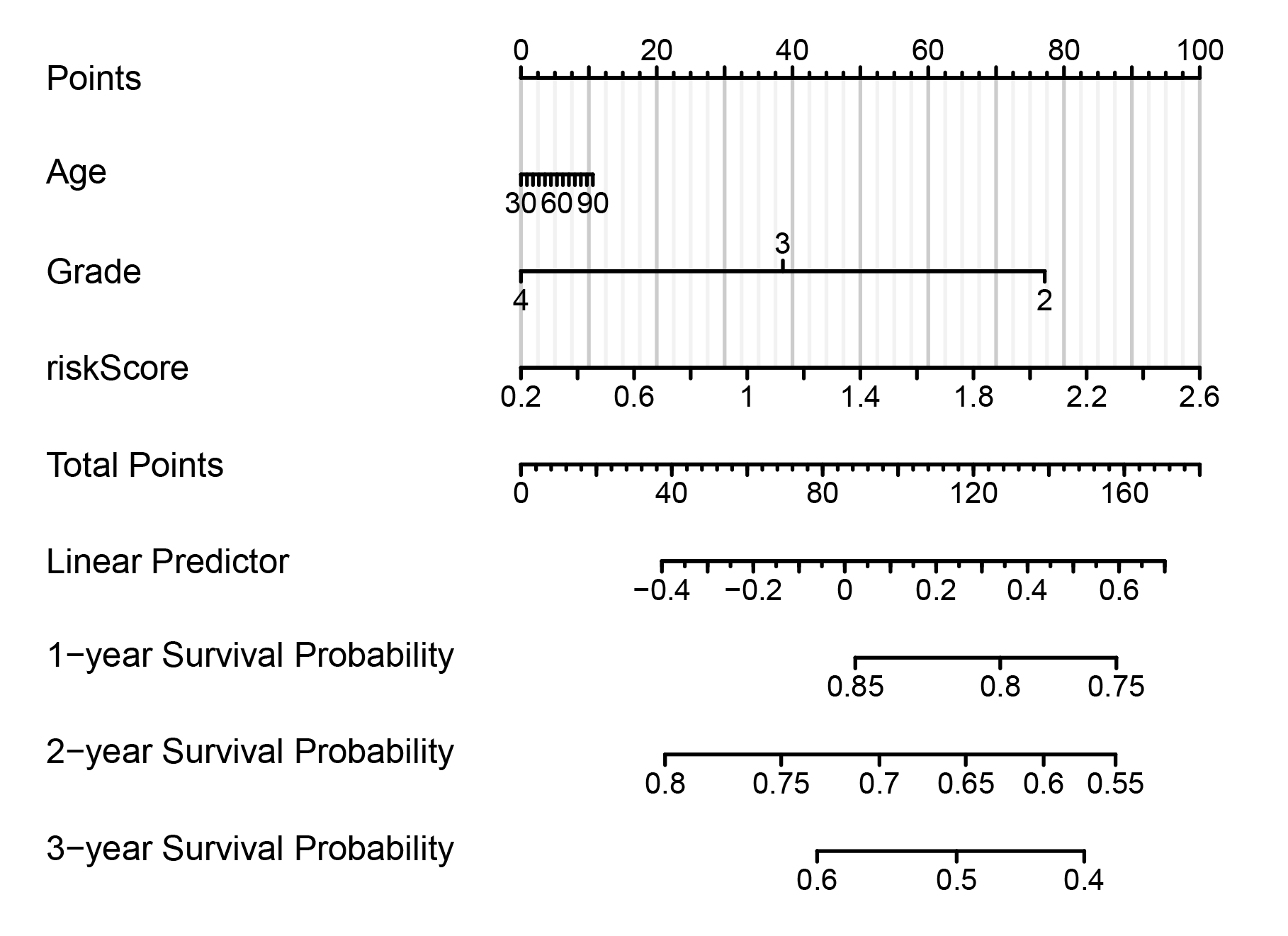

Supplement: Supplementary file 8 [file Image5.TIF]
